# Contemporary review of stress echocardiography workforce within the UK: an EVAREST/BSE NSTEP study

**DOI:** 10.1186/s44156-025-00088-x

**Published:** 2025-10-10

**Authors:** James Willis, Casey L. Johnson, Samuel Krasner, William Woodward, Annabelle McCourt, Cameron Dockerill, Katrin Balkhausen, Badrinathan Chandrasekaran, Attila Kardos, Nikant Sabharwal, Soroosh Firoozan, Rizwan Sarwar, Roxy Senior, Rajan Sharma, Kenneth Wong, Maria Paton, Jamie O’Driscoll, David Oxborough, Keith Pearce, Shaun Robinson, Adora Mo Wah Yau, Daniel X. Augustine, Paul Leeson

**Affiliations:** 1 Royal United Hospitals, Bath, UK; 2 Cardiovascular Clinical Research Facility, Division of Cardiovascular Medicine, John Radcliffe Hospital, University of Oxford, Oxford, OX3 9DU UK; 3 Barts and the London School of Medicine and Dentistry, Queen Mary University of London, London, UK; 4 Kings College London, London, UK; 5 Royal Berkshire Hospital, Reading, UK; 6 Great Western Hospital, Swindon, UK; 7 Department of Cardiology, Translational Cardiovascular Research Group, Milton Keynes, and Faculty of Medicine and Health Sciences, Milton Keynes University Hospital NHS Foundation Trust, University of Buckingham, Buckingham, UK; 8 Oxford University Hospitals NHS Foundation Trust, Oxford, UK; 9 Wycombe Hospital, High Wycombe, UK; 10 Northwick Park Hospital, London, UK; 11 Department of Cardiology, St George’s University Hospitals NHS Foundation Trust, London, UK; 12 Lancashire Cardiac Centre, Blackpool Teaching Hospitals NHS Foundation Trust, Blackpool, UK; 13 University of Leeds, Leeds, UK; 14 Leeds Teaching Hospitals Trust, Leeds, UK; 15 Diabetes Research Centre, College of Life Sciences, University of Leicester, Leicester, UK; 16 Liverpool John Moores University, Liverpool, UK; 17 Manchester University NHS Foundation Trust, Manchester, UK; 18 Imperial Healthcare NHS Trust, London, UK; 19 Department of Life Sciences, Manchester Metropolitan University, Manchester, UK; 20 Department for Health, University of Bath, Bath, UK

**Keywords:** Ischaemic heart disease, Workforce, Stress echocardiography

## Abstract

**Background:**

Stress echocardiography is a key imaging modality for assessing coronary artery disease in the UK. Traditionally, stress echo services were led by consultant cardiologists, but evolving workforce models have increased the involvement of cardiac physiologists and scientists. This study, as part of the National Review of Stress Echocardiography Practice (BSE N-STEP), aimed to evaluate current stress echo workforce structures and test outcomes across a group of UK hospitals to inform future workforce planning.

**Results:**

Data were analysed from 8506 stress echocardiograms, conducted between September 2020 and June 2023 across 34 UK hospitals. Based on the supervising workforce, stress echocardiograms were allocated into either a doctor-led (DL) or cardiac physiologist/scientist and nurse-led (CNL) model. 56.9% of stress echocardiograms were DL, while 42.7% were conducted under a CNL model. Physiologists/scientists were the most frequently involved staff (81.9%). The primary indication for stress echocardiography was ischaemia evaluation (89.4%). Dobutamine stress echocardiography was more common in DL services (63.0 vs. 56.3%, p < 0.001), while CNL services performed more exercise stress echocardiography (42.8 vs. 36.4%, p < 0.001). Test positivity rates were similar between DL and CNL models (17.1 vs. 17.7%, p = ns), though the CNL group had a lower complication rate (2.2 vs. 5.3%, p < 0.001). Reporting of stress echocardiograms remained consultant-led in 82% of cases, but physiologist/scientist-led reporting showed an increase over time. Training was primarily provided to registrars/fellows (60.2%), with physiologist/scientist trainees accounting for 32.4%.

**Conclusions:**

This study provides a contemporary overview of stress echocardiography workforce models in the UK, highlighting the increasing role of cardiac physiologists and scientists in supervising and reporting stress echocardiography. Despite these shifts, consultant cardiologists remain central to stress echo reporting. The findings support the integration of multidisciplinary workforce models to enhance service efficiency. These insights will aid in future workforce planning and training strategies to optimise stress echocardiography service provision across the NHS.

**Supplementary Information:**

The online version contains supplementary material available at 10.1186/s44156-025-00088-x.

## Introduction

Stress echocardiography remains the most common imaging modality for investigation of coronary artery disease in the UK and cost-effective delivery of this service is important. In 2014, the British Society of Echocardiography (BSE) surveyed 120 centres and found a predominant doctor-led stress echocardiography workforce model, comprising consultant cardiologists and trainees [1]. Since then, workforce delivery models have evolved significantly across cardiology practice and recent single centre studies indicate stress echocardiography practice may also be changing [2–4].

Initiatives like'Modernising Scientific Careers'[5, 6] have promoted the role of physiologists and scientists as lead service providers. Such workforce models offer enhanced clinical capacity and flexibility without compromising patient care [7, 8]. Recent European stress echocardiography guidelines now also provide clear frameworks for training and competency [9].

Within the UK, the National Review of Stress Echocardiography Practice (BSE N-STEP) [10] has been collecting comprehensive data on stress echocardiography practice, workforce models, and diagnostic performance across 34 hospitals. Using data from N-STEP, we now report the current predominant workforce models for stress echocardiography delivery within the UK, including an analysis of the patient referral patterns, outcomes and variation across type and size of hospital. This data provides unique contemporary data, at a whole health service level, to support workforce planning, education, and the dissemination of best practices.

## Methods

### Study population

The EVAREST study (NCT03674255) is a prospective, multi-centre observational study into the use of and outcomes from stress echocardiography studies conducted across multiple UK NHS sites. The early results of this study, which examined the long-term outcomes of stress echo activity, have recently been published [10]. BSE N-STEP is phase three of the EVAREST study, which has recently been completed. This last phase of the study recruited patients who were referred for stress echocardiography for any indication from September 2020 to June 2023. All participants were over 18 years of age and provided written informed consent. In a centralised database, the individual centres documented details about the stress echocardiogram study itself, including the primary indication for testing, stress modality, workforce, and study outcome. The full study design has already been described [11]. NHS ethical approval for the study was granted as part of phase 3 of the EVAREST project (Ref: 14/SC/1437). This study was conducted in accordance with the Declaration of Helsinki.

### Stress echo and clinical information

Each test was conducted according to the standard protocol of the individual hospitals. For example, the use of contrast and atropine was dictated by the individual hospitals and recorded post-test. The baseline characteristics of the participants were documented, including anthropometric data, gender, and resting heart rate and blood pressure. This was self-reported by each respective test centre and included information on risk factors and any previous cardiac testing. Further information regarding the immediate outcome of the test was also recorded, along with any complications that may have occurred. All data was obtained from the electronic patient record of the hospital and recorded on an electronic database (Castor EDC, Amsterdam, Netherlands).

### Workforce data

The workforce involved with each stress echocardiography was documented in two distinct ways. Firstly, the staff present during the test were identified from a pick list. Options available included consultant cardiologist, cardiology registrar/fellow, cardiac physiologist/scientist, nurse, healthcare assistant, student or other. If other were selected, further details on the staffing group would be required as a free text entry. Given the binary data entry, as each option could only be selected once, it was not possible to discern if multiple of the same role were present, unless specified elsewhere. When only one staff member was listed for the test, confirmation was sought to clarify the staffing arrangements.

Secondly, each centre also reported the respective roles that staff undertook during the delivery of the stress echocardiography encounter. This was broken down into the test supervision and test reporting phases. This included identifying the staff group acting as the primary operator—the individual responsible for performing the scan,—the supervisor—the individual responsible for managing the test, and finally, the individual responsible for reporting the stress echocardiogram results.

Using the self-reported ‘supervising’ staff data, studies were broadly categorised as doctor-led (DL) (which included both senior consultants and doctors in training) or cardiac physiologist/scientist and nursing-led (CNL). The same process was performed based on the individuals reporting the test, with further analysis of each clinical group.

Where the staff (e.g., consultant) listed as reporting the test differed from those present in the room (e.g., physiologist/scientist & nurse), the individual was deemed to be acting as a “reader” where reporting of the test is undertaken and authorised by a senior clinician outside of the group directly supervising the test and without any influence on the dynamic test situation [2].

Where the consultant was listed as being present within the room, but the report was performed by another (e.g., the registrar), this was considered dual reporting, where the results were interpreted in conjunction with the consultant lead and the noted staff member. The potential for training during the stress echocardiogram was also recorded, along with the recipient and the staff supervising the training. This was designed to better understand the capacity and scope of training for the future workforce and the scope of staff present to engage with stress echo services.

### Statistical testing

Patient demographics were reported using mean, standard deviation, and median (interquartile range (IQR)) values where appropriate, as well as percentages for categorical variables. Normality was assessed using the Sapiro-Wilk test. Each hospital was categorised based on the number of hospital beds reported by NHS England [12] into one of four different groups (< 600, 600–799, 800–1000 and > 1000 beds). This was used to measure the impact of hospital size on potential service delivery and training. In addition, hospitals were broadly characterised as either a Tertiary/Teaching centre, or a District General Hospital (DGH).

Comparisons between groups were made using Mann–Whitney or Chi-square testing, where appropriate. Binary logistic modelling was used to identify key variables impacting study outcomes. Multinomial logistic regression analysis examined the relationship between test supervision models (Consultant, Registrar/Fellow, Physiologist/Scientist, and Nurse) and predictor variables, including patient demographics and documented risk factors, including smoking status and regional wall motion abnormalities at rest. Participants with missing demographic data were included in the study, and missing data points were not interpolated. A P-value < 0.05 was considered significant.

## Results

### Study group demographics

**Fig. 1:**
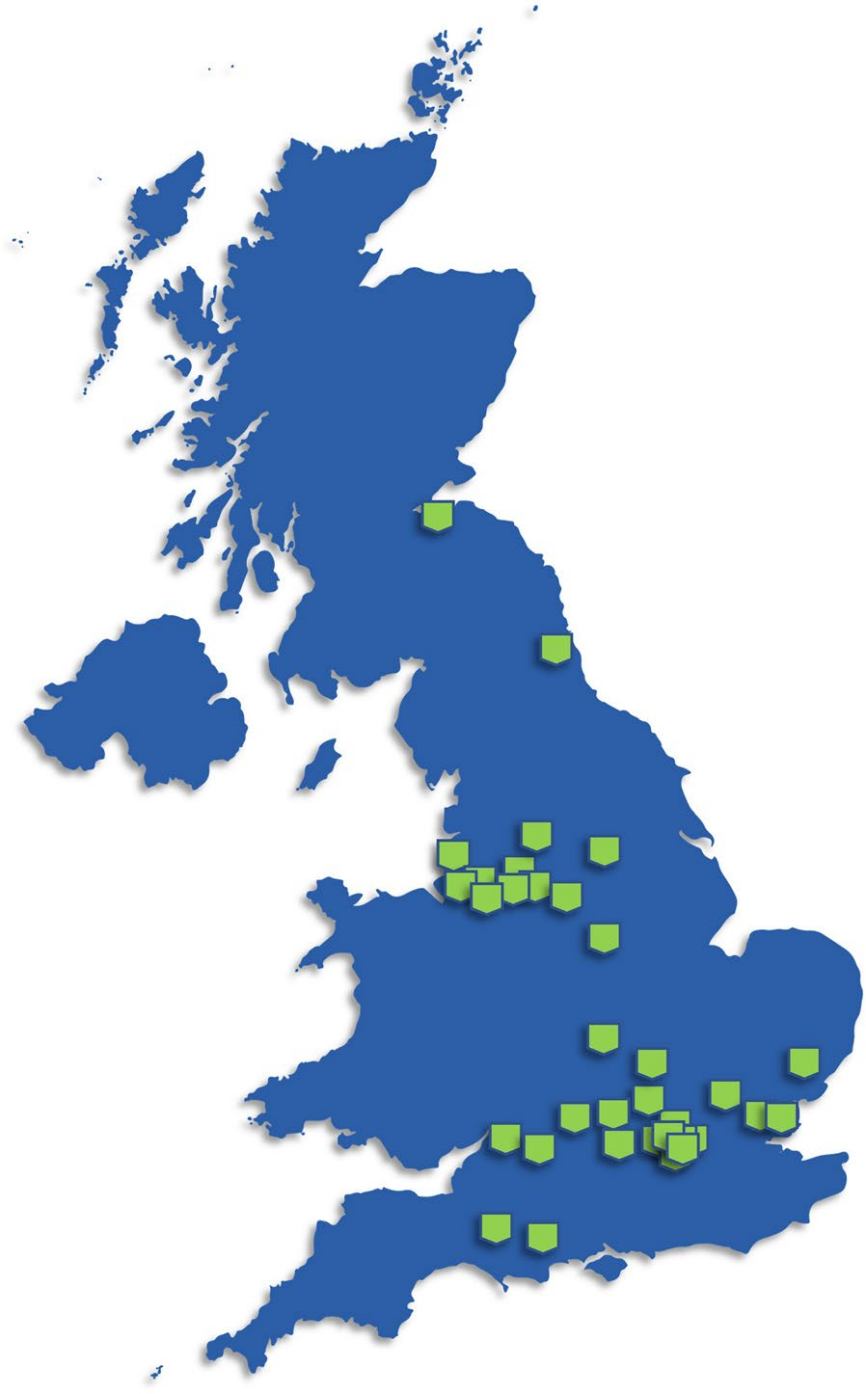
Location of recruiting NSTEP Hospitals

**Fig. 2:**
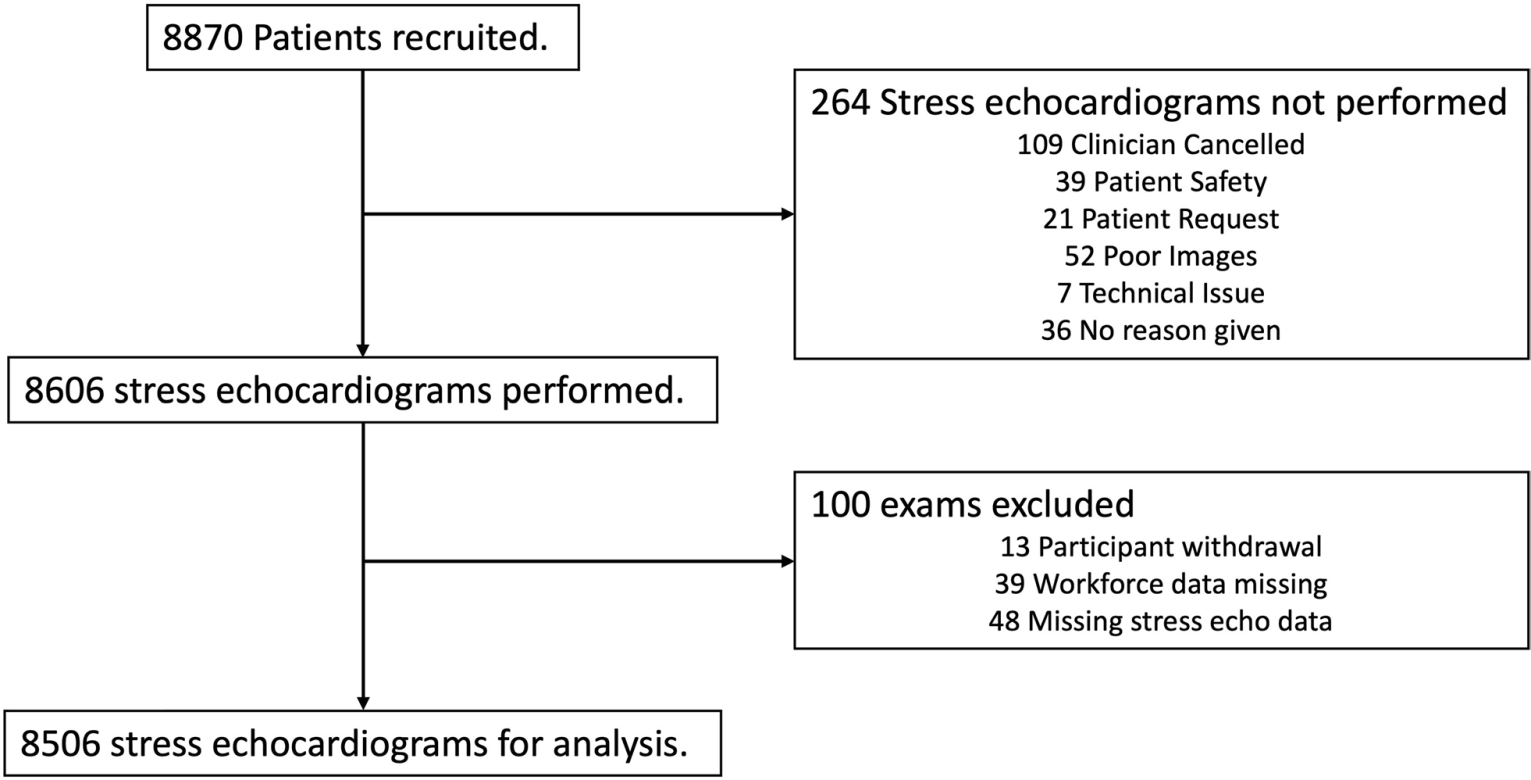
Recruitment flow chart from September 2020 to June 2023

From September 2020 to June 2023, 8870 patients were recruited into the N-STEP database from 34 different hospital sites shown in Fig. 1. Within the cohort 56% of hospitals were identified as DGH (secondary care) with the remaining 44% as Teaching/Tertiary hospitals. Figure 2 shows an inclusion and exclusion flow chart for this study with 264 (3%) tests not undertaken and excluded from the final group analysis. 8606 then proceeded to have a stress echocardiogram performed. A further 100 studies were excluded post stress echocardiogram due to either patient withdrawal, or key data missing. This left a total of 8506 potential participants eligible for analysis.

Table 1 reports the complete patient demographics. The median age was 66 years (IQR 57–74), and 58.3% of the participants were male. Table 2 shows the indications recorded for stress echocardiography, with the majority, 7602 (89.4%), undertaken to assess ischaemic heart disease. This was identified across all 34 hospitals. Twenty-two Hospitals (65%) performed studies for valvular heart disease, with 17 (50%) performing studies for myocardial viability within the study window.

**Table 1:** Patient demographics at time of stress echocardiogram

	Total Group n = 8506	
Male (%)	4957	58.3%
Median age (years) (IQR)	66	(57–74)
Median BMI (Kg/m2) (IQR)	28.56	(25–32)
Smoking		
Non-Smoker (%)	4350/8091	51.1%
Ex Smoker (%)	2785/8091	32.7%
Current Smoker (%)	956/8091	11.2%
Hypertension (%)	4440/8469	52.2%
Hypercholesterolaemia (%)	3756/8469	44.2%
Diabetes mellitus (%)	1829/8469	21.5%
Family history of premature CAD	2613/8469	30.7%
Previous MI	1419/8468	16.7%
Previous PCI	1649/8468	19.4%
Previous CABG	558/8468	6.6%
Peripheral vascular disease	175/8468	2.1%

**Table 2:** Total group Indication for Stress Echocardiography

	Total group n = 8449	%
Exclude ischaemic heart disease	7574/8449	89.4%
Myocardial viability assessment	80/8449	0.9%
Valve assessment	147/8449	1.7%
Left ventricular outflow tract obstruction assessment	28/8449	0.3%
Diastology assessment	1/8449	0.01%
Pre-transplant/pre-operation assessment	250/8449	2.9%
Other (please specify)	355/8449	4.2%
Inconclusive prior cardiac testing	14/8449	0.2%

### Workforce models—whole study population

On average, two staff members undertook the stress echo test. A physiologist/scientist was present in 6965 (81.9%) tests and was the most frequent staff member in the study cohort. A consultant cardiologist was listed as present in 4085 (48%), a registrar/fellow in 2451 (28.8%) and a nurse in 2972 (34.9%). These staffing groups combined in various ways to provide the clinical cover and expertise needed to undertake the test, with roles being interchangeable based on training needs if appropriate. Across all sites, 4839 (56.9%) studies were performed by a DL service model, and 3636 (42.7%) by a CNL service model. 3863 (79.8%) of studies within the DL model were supervised by a consultant cardiologist and 976 (20.2%) by a registrar. Within the CNL group, 2861 (78.7%) of studies were led by a physiologist/scientist, and 775 (21.3%) were nurse-led.

The median number of staff present in the DL model was 2 (IQR 2–3) with the most common combination being a consultant and a physiologist. In contrast, the CNL model also had a median of 2 staff (IQR 1–3) per test, with the most common combination being two physiologist/scientist staff.

### Workforce models—centre level

Of the 34 centres recruiting into NSTEP, 28 (82%) ran both a DL and CNL-supervised stress echo service, while the remaining six ran DL services only. No centre ran a CNL service in isolation without cardiologist involvement. In 12 (43%) centres more stress echocardiograms were supervised by CNL than DL clinics. A CNL service model was more likely to be used in larger hospitals with > 1000 beds, compared to both medium sized hospitals with 600 to 799 beds (OR 0.77 95% CI 0.66–0.88, p < 0.001) and hospitals with 800 to 1000 beds (OR 0.55 95% CI 0.46–0.64, p < 0.001). However, a CNL service was most common in smaller hospitals with < 600 beds, with a higher likelihood than in larger centres with > 1000 beds (OR 2.91 95% CI 2.54–3.34, p < 0.001).

### Workforce models and patient characteristics

Logistic regression was conducted to investigate the effects of age, sex, cardiac risk factors, and hospital bed size on the likelihood of test supervision being either DL or CNL. The CNL group saw younger patients (OR 0.99 95% CI 0.98–0.99, p < 0.001) but with a higher cardiovascular risk profile based on the presence of hypercholesteremia (OR 1.30 95% CI 1.16–1.44, p < 0.001) and diabetes (OR 1.28 95% CI 1.13–1.46, p < 0.001), although a higher rate of never-smokers (53.2 vs. 49.6%, p = ns). The CNL service model was less likely to see patients with a previous family history of premature coronary disease (OR 0.71 95% CI 0.62–0.79, p < 0.001) or resting regional wall motion abnormalities (OR 0.64 95% CI 0.54–0.76, p < 0.001) compared to the DL group.

### Workforce and stress echocardiography study characteristics

Table 3 provides complete patient demographics for both the DL and CNL groups. The proportion of patients seen for ischaemic heart disease tests was similar between DL and CNL clinics (89.1 vs 89.7%, p = ns), but CNL services performed more viability (0.8 vs 1.2%, p = 0.04) and pre-op studies (2.6 vs 3.4%, p = 0.03). Compared to the CNL group, DL services more commonly performed dobutamine stress echo (DSE) (63.0 vs 56.3%, p < 0.001), whilst the CNL group performed more exercise stress echo (ESE) (42.8 vs 36.4%, p < 0.001) and specifically more treadmill exercise (32.8 vs 25.1%, p < 0.001) whilst rates of bicycle exercise were similar (11.2 vs 10% p = ns) (see supplementary Tables 4 and 5). Stress echo positivity rates were similar across DL and CNL groups (17.1 vs 17.7%, p = ns), as were inconclusive/abandoned tests (3.8 vs 3.6%, p = ns). There was a lower rate of reported complications in CNL studies compared to DL studies (2.2 vs 5.3%, p < 0.001).

**Table 3:** Patient Demographics Separated by Test Supervision Model

	Doctor Led (DL) (n = 4839, 56.9%)		Cardiac Physiologist/Scientist or Nurse Led (CNL) (n = 3636, 42.7%)		*P*
	n		n		
Male (%)	2762	57.1	2179	59.9	ns
Median age (years) (IQR)	66	57—74	65	56—73	< 0.001
Median BMI (Kg/m2) (IQR)	28.3	25.1—32.4	28	25.3—32.5	< 0.001
Smoker					
Non-smoker (%)	2400	49.6	1936	53.2	
Ex smoker (%)	1578	32.6	1194	32.8	0.007
Current smoker (%)	513	10.6	441	12.1	< 0.001
Hypertension (%)	2425	50.1	1995	54.9	ns
Hypercholesterolaemia (%)	1916	39.6	1828	50.3	< 0.001
Diabetes mellitus (%)	911	18.8	905	24.9	< 0.001
Family history of premature CAD	1454	30	1153	31.7	< 0.001
Peripheral vascular disease	99	2	75	2.1	ns
Previous MI	724	15	693	19.1	ns
Previous PCI	835	17.3	809	22.2	ns
Previous CABG	283	5.8	274	7.5	ns
Resting RWMA	637	13.2	319	8.8	< 0.001
Hospital bed size					
< 600 Beds	638	13.2	1049	28.9	< 0.001
600–799 beds	1074	22.2	485	13.3	< 0.001
800–1000 beds	1364	28.2	1125	30.9	< 0.001
> 1000 beds	1763	36.4	977	26.9	–

### Workforce and reporting

**Fig. 3:**
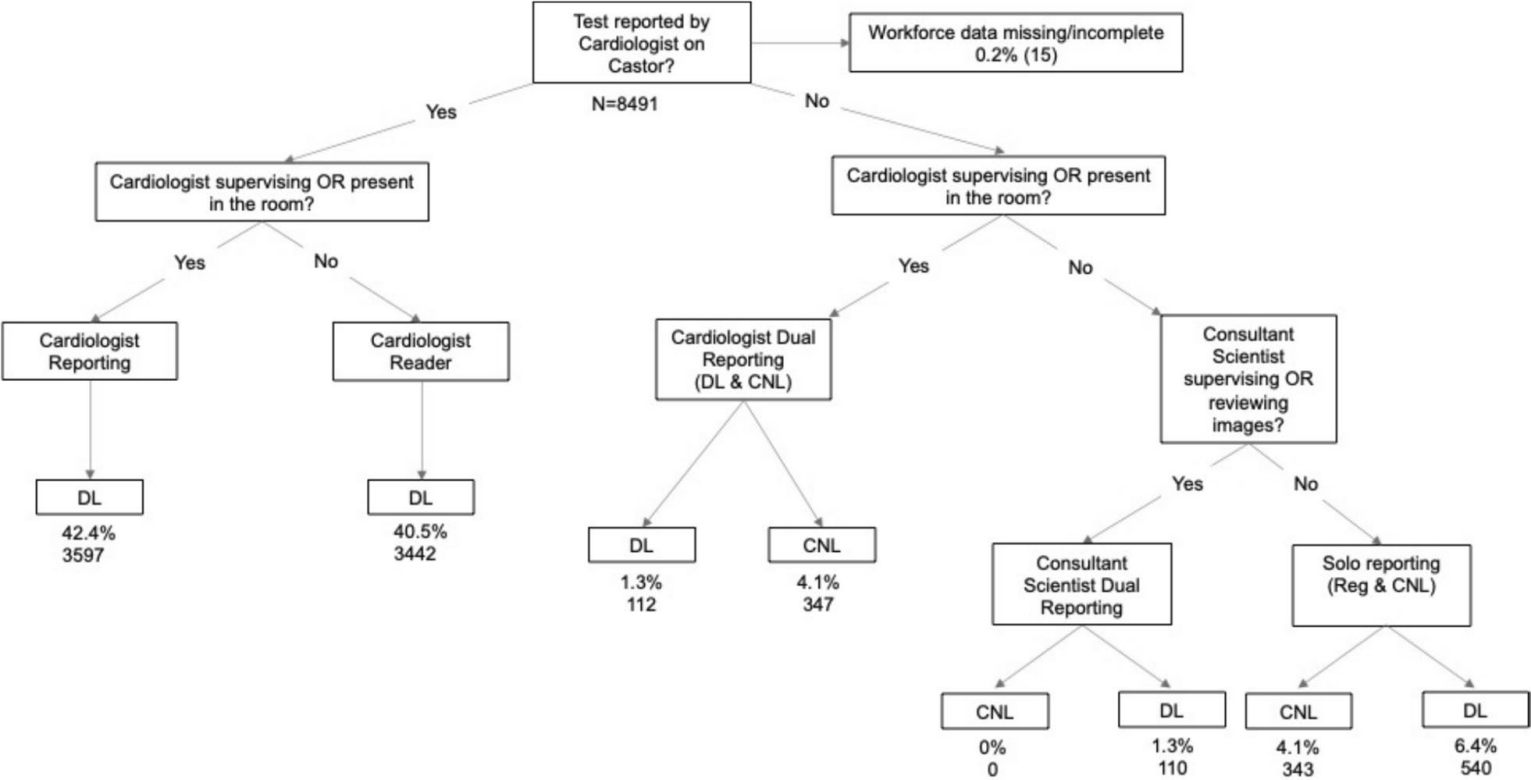
Reporting structure identified for Doctor Led (DL) and Cardiac Physiologist/Scientist and Nurse Led (CNL) reporting of stress echo results. Results show the number of stress echocardiograms and an overall percentage

**Fig. 4:**
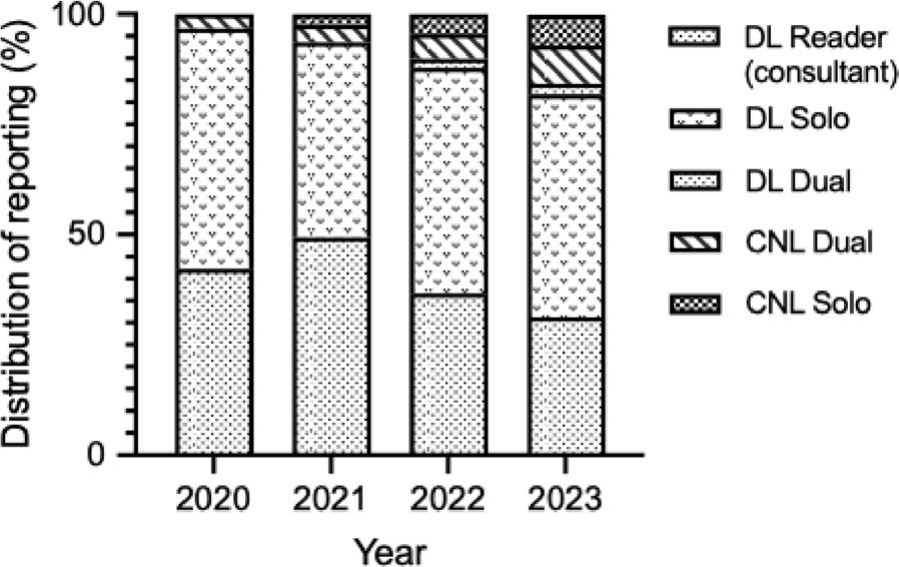
Doctor Led (DL) and Cardiac Physiologist/Scientist and Nurse Led (CNL) stress echocardiography reporting as a percentage change, year on year. This shows how the CNL reporting pathway has increased over the duration of the project

Figure 3 demonstrates the different reporting combinations. Cardiologists reported the highest proportion of stress echocardiograms (n = 7039, 82%) with consultant reporting in all 34 hospitals being the most common model. A cardiologist was both present and reported the stress echocardiogram in 3597 (42.4%) studies. In the remaining 3442 (40.5%) studies, seen in 22 individual hospitals, a cardiologist reported and authorised the test, while test supervision was undertaken by other staff members. 445 studies had dual reporting, with either a registrar/fellow, physiologist/scientist, or nurse reporting the study alongside a consultant cardiologist. Within the CNL group, 110 studies were dual reported with a consultant clinical scientist, and 343 studies were reported independently, with 98.8% reported by physiologists/scientists. 16 individual hospitals reported a physiologist/scientist reporting model. Registrars/fellows independently reported 540 studies across 12 (35.3%) hospitals. Figure 4 demonstrates a year-on-year increase in physiologist/scientist solo or dual reporting of studies, with 13.8% reported studies in 2023, without a change in other healthcare reporting groups.

### Training during stress echocardiography

20 of the 34 (58.8%) recruiting centres reported training during stress echo clinics involving 1729 studies (20.3%). 1040 studies (60.2%) involved a cardiology registrar/fellow as the trainee, 560 (32.4%) involved a physiologist/scientist, 71 (4.1%) a nurse and 18 (1.0%) a consultant cardiologist. A further 40 (2.3%) studies involved other staff groups or those on physiologist/scientist university training schemes. The roles undertaken during this training included test management (supervision), image acquisition, and direct observation of the test. Some studies involved training a cardiology registrar/fellow and a physiologist/scientist during the same test. Training rates compared based on hospital bed size indicated staff training was most likely to occur in a hospital of 800 beds or more (p < 0.001) and within the registrar/fellow group.

## Discussion

This study provides the first contemporary data on staff workforce models currently employed to deliver real-world stress echocardiogram services within the UK. A multi-professional workforce is being used to meet the demands of stress echocardiography services, with a prominent role for cardiac physiologists and scientists, who have a broad capability, including delivery of pharmacological stress testing. A continued major role for consultant cardiologists in reading and reporting the test results has also been identified.

Previously, it has been demonstrated that stress echocardiography services can be delivered by a broad workforce beyond the traditional cardiologist-led model, including nurses, scientists, consultant scientists, and doctors in training. The expanded scope for non-medical professionals has been well-documented, with competency frameworks supporting structured training and accreditation [13, 14]. Single-centre studies affirm the safety of physiologist-led stress echocardiography with adequate support and training [2–4, 15]. We now present data that these models are being implemented into real-world practice within the UK. In this analysis of – 8500 stress echocardiogram exams, physiologists/scientists were a significant staff group, with nearly half of all stress echocardiograms conducted under non-medical supervision.

Stress echocardiography reporting followed varied models. Although 94.6% of studies classed as DL were cardiologist-reported, for 40.5% of these the cardiologist was the reviewer of the study and not present during the testing. This potentially reflects a balance between greater efficiency with CNL stress studies but an expectation for ongoing expert cardiologist oversight [2, 16, 17]. Compared to the survey results from 2014, there has also been an increase in independent stress echocardiography reporting by registrars/fellows and non-medical clinicians. Future work could consider further the potential financial savings accrued by this service model.

Ischaemic testing remained the primary indication, consistent with previous UK [1] and multi-centre studies [18]. Single centre studies such as Ntoskas et al. [4] have reported 93.7% of tests in physiologist-led clinics assessing ischaemia and this appears to be being replicated across multiple hospitals. Despite recruitment allowing for all types of stress echo, the number of studies performed for valvular heart disease assessment was very low. This may reflect a selection bias related to recruitment practice in each centre. In earlier phases of the study only patients undergoing ischaemia testing were recruited and recruitment practice may not have altered. Nevertheless, further work to establish the true extent to which stress echo is being used for valvular assessment will be of value for development of future workforce training plans.

The predominant use of stress echo for ischaemia testing likely explains the key observation that DSE was the most common stressor, including in the CNL group. Although the CNL group conducted more ESE than DL services, the CNL services predominantly used dobutamine stressors. This potentially reflects institutional preferences for different stress pathways as DSE and ESE use ranged from 0–100% across the CNL group. This pathway stratification may also help explain the differences in complication rates seen between the two groups. Prior studies within the UK centres performing physiologist/scientist-led stress echo reported varied preferences for either ESE dominance [69.2% [3] and 91.5% [2]] versus DSE [98% [4]]. Italian and Austrian national surveys similarly showed dobutamine as the most used stressor (85.4 [18] and 91% [19]).

Despite these changes in workforce utilisation, training appears to be still predominantly focused on registrars/fellows, particularly in larger hospitals, and is likely to follow already established medical training programmes. Although details of training activities were not always available, it is reasonable to assume registrar-led clinics mirrored consultant-led settings in patient demographics. Nevertheless, 82% of centres ran combined DL and CNL services, reflecting recognition of hybrid model workforces and training benefits [20]. Compared to 2014, staffing diversity increased, with models like the physiologist-led Rapid Access Chest Pain Stress Echocardiography clinic [2] facilitating efficient diagnostic image acquisition for cardiologist review. Future work within this area must consider the impact and cost involved in establishing both DL and CNL services, through the framework of accreditation pathways such as those offered by the BSE.

This study has limitations. Firstly, it is difficult to determine the number of operators available at each site and also the level of operator experience with for example BSE accreditation, as this was not captured in the workforce data. Secondly, some assumptions are made that studies were reported on the day of the test rather than during a separate reporting session, which was not captured in the study data and could impact the efficiencies claimed within the study. Thirdly, whilst the larger EVAREST study has permission to follow-up patients up to 10 years post stress echo, this study reports only the immediate test implications. Despite this, the long term follow-up of patients in both DL and CNL clinics has previously been documented [4, 15]. Fourthly, the volume of data collected may not represent the activity undertaken by the centres in question, and not all studies started recruiting at the same time. This means that some sites have contributed more significantly than others. Fifthly, the prospective observational study design may lead to changes in patient recruitment patterns over the course of the study. Finally, although providing contemporary information on workforce practice there is no historical study similar in design to give true equivalent comparator data and any comparisons are based on previous single centre or survey-based datasets.

## Conclusion

This study provides the latest data on the delivery of stress echocardiography services within the NHS. Unique to this study is its design, utilising the EVAREST study network, involving a very large study population, representative of real-world UK practice. This provides multicentre information across a range of test modalities and hospital settings. The UK stress echo workforce no longer appears to reflect the working patterns described in previous position statements and survey studies. While review and reporting continue primarily to be undertaken by cardiologists, current delivery of stress echocardiography involves a high proportion of non-medical-led services. This includes test supervision as well as support and is being performed across all indications and stress modalities. These practices are likely to be introducing significant efficiencies and cost benefits to delivery of stress echocardiography within the NHS.

## Supplementary Information

Supplementary Material 1.

## Data Availability

The data underlying this article will be shared on reasonable request to the corresponding author.
