# Supplementary material for: Contemporary review of stress echocardiography workforce within the UK: an EVAREST/BSE NSTEP study
Source: Echo Res Pract. 2025 Oct 10;12:22. doi: 10.1186/s44156-025-00088-x (PMC12512890; doi:10.1186/s44156-025-00088-x)
Supplement: Supplementary file 1 — Supplementary Material 1. [file 44156_2025_88_MOESM1_ESM.docx]

**Supplemental Tables**

Table 4 Indications for stress echo separated by Test Supervision Model

|  | Doctor led (DL) | | Cardiac physiologist/scientist or nurse led (CNL) | |  |
| --- | --- | --- | --- | --- | --- |
|  | n | % | n | % | *p* |
|  |  |  |  |  |  |
| Exclude Ischaemic Heart Disease | 4312 | 89.1% | 3262 | 89.7% | ns |
| Myocardial Viability Assessment | 37 | 0.8% | 43 | 1.2% | 0.046 |
| Valve Assessment | 89 | 1.8% | 58 | 1.6% | ns |
| LV Outflow Tract Obstruction Assessment | 22 | 0.5% | 6 | 0.2% | 0.022 |
| Diastology Assessment | 1 | 0.002% | 0 | 0.0% | - |
| Pre-Transplant/ Pre-Operation Assessment | 127 | 2.6% | 123 | 3.4% | 0.037 |
| Inconclusive prior cardiac testing | 10 | 0.2% | 4 | 1.0% | ns |

Table 5 Stressor used during stress echo separated by Test Supervision Model

|  | Doctor Led (DL) | |  | Cardiac Physiologist / Scientist or Nurse Led (CNL) | |  |  |
| --- | --- | --- | --- | --- | --- | --- | --- |
|  | n | % |  | n | % |  | *p* |
|  |  |  |  |  |  |  |  |
| (Pharmacological) Dobutamine Stress Echo | 3047 | 63.00% |  | 2048 | 56.30% |  | <0.001 |
| Exercise Stress Echo | 1763 | 36.40% |  | 1555 | 42.80% |  | <0.001 |
| Treadmill Exercise | 1214 | 25.10% |  | 1192 | 32.80% |  | <0.001 |
| Bicycle Exercise | 541 | 11.20% |  | 365 | 10.00% |  | 0.092 |
| Pacemaker Stress Echo | 16 | 0.30% |  | 17 | 0.50% |  | 0.317 |
| Myocardial Contrast Echo (Perfusion) | 5 | 0.10% |  | 11 | 0.30% |  | 0.037 |
